# Supplementary material for: Immersive Technologies Targeting Spatial Memory Decline: A Systematic Review
Source: Biomedicines. 2025 Aug 29;13(9):2105. doi: 10.3390/biomedicines13092105 (PMC12466985; doi:10.3390/biomedicines13092105)
Supplement: Supplementary file 1 [file biomedicines-13-02105-s001.zip › biomedicines-3800383-supplementary.pdf]

Table S1. Risk of Bias Using the JBI Critical Appraisal Checklists [28].

Type of study	Author (year)	Q1	Q2	Q3	Q4	Q5	Q6	Q7	Q8	Q9	Q10	Q11	Q12	Q13	Overall score
**Cross-Sectional Studies**	Andac et al., 2024 [30]	Y	Y	N	Y	Y	Y	Y	Y						7
	Castegnaro et al., 2022 [33]	Y	Y	Y	Y	Y	Y	Y	Y						8
	Castegnaro et al., 2023 [34]	Y	Y	Y	Y	Y	Y	Y	Y						8
	Castillo Escamilla et al., 2023 [35]	Y	U	Y	Y	N	N	Y	Y						5
	Cammisuli et al., 2024 [32]	Y	Y	Y	Y	N	N	Y	Y						6
	Chatterjee & Moussavi, 2025 [36]	Y	Y	Y	Y	Y	Y	Y	Y						8
	Da Costa et al., 2021 [38]	Y	Y	Y	Y	N	N	Y	Y						6
	De Silva et al., 2023 [39]	U	Y	Y	Y	N	N	Y	Y						5
	Diersch et al., 2021 [40]	Y	Y	Y	Y	Y	Y	Y	Y						8
	Goodroe et al., 2025 [42]	Y	Y	Y	Y	Y	Y	Y	Y						8
	Hanert et al., 2024 [43]	Y	Y	Y	Y	Y	Y	Y	Y						8
	Hilton et al., 2020 [45]	U	Y	N	Y	N	N	Y	Y						4
	Kalantari et al., 2024 [46]	U	Y	N	Y	N	N	Y	Y						4
	Koike et al., 2024 [48]	Y	Y	N	Y	N	N	Y	Y						5
	Lowry et al., 2020 [51]	Y	Y	Y	Y	Y	Y	Y	Y						8
	McAvan et al., 2021 [52]	Y	Y	N	Y	N	N	Y	Y						5
	McCracken et al., 2025 [53]	Y	Y	Y	Y	Y	Y	Y	Y						8
	Newton et al., 2024 [54]	Y	Y	Y	Y	Y	Y	Y	Y						8
	Noguera et al., 2020 [55]	Y	Y	N	Y	Y	U	Y	Y						6
	Rinne et al., 2022 [60]	Y	Y	N	Y	Y	Y	Y	Y						7
	Wiener et al., 2020 [68]	U	Y	Y	Y	Y	Y	Y	Y						7
**Quasi-experimental Studies**	Chen et al., 2024 [37]	Y	Y	Y	Y	N	Y	Y	N/A	Y					7
	Ladyka-Wojcik et al., 2021 [49]	Y	Y	Y	Y	Y	Y	Y	Y	Y					9
	Lokka & Çöltekin, 2019 [50]	Y	Y	Y	Y	Y	Y	Y	Y	Y					9
	Oliver et al., 2024 [56]	Y	Y	Y	Y	Y	Y	Y	Y	Y					9
	Shayman et al., 2024 [61]	Y	Y	Y	Y	Y	Y	Y	Y	Y					9
	Stramba-Badiale et al., 2024 [62]	Y	Y	Y	Y	N	Y	Y	Y	Y					8
	Sunami et al., 2025 [63]	Y	N	Y	Y	Y	Y	Y	Y	Y					9
	Tuena et al., 2024 [64]	Y	Y	N	Y	Y	Y	Y	U	Y					8
	Tuena et al., 2024 [65]	Y	Y	Y	Y	Y	Y	Y	Y	Y					9
	Wen et al., 2023 [67]	Y	N	Y	Y	Y	Y	Y	Y	Y					8
	Zuo & Zhou, 2024 [70]	Y	Y	Y	Y	Y	Y	Y	Y	Y					9
**Randomized Controlled Trials (RCT)**	Amaefule et al., 2023 [29]	Y	N	Y	N	N	Y	N	Y	Y	Y	Y	Y	Y	9
	Qiu et al., 2024 [59]	Y	N	Y	N	N	Y	N	Y	Y	Y	Y	Y	Y	9
	Xu et al., 2025 [69]	Y	Y	Y	Y	Y	Y	Y	Y	Y	Y	Y	Y	Y	13

**Diagnostic Test Acuracy Studies**	Bayahya et al., 2021 [31]	N	N	Y	U	N/A	Y	U	U	Y	Y				4
	Fu et al., 2022 [41]	Y	N	Y	U	N/A	Y	U	Y	Y	Y				6
	Kim et al., 2023 [47]	N	Y	Y	U	N/A	Y	U	Y	Y	Y				6
	Park, 2022 [57]	N	N	Y	Y	N	Y	Y	Y	Y	Y				7
	Puthusseryppady et al., 2022 [58]	N	N	Y	U	N/A	Y	U	Y	Y	Y				5
**Cohort Study**	Hanyu et al., 2024 [44]	Y	Y	Y	Y	Y	Y	Y	N	Y	N	Y			9

* Abbreviations (in alphabetical order): Y = Yes, U = Unclear, N= No; N/A= No Applicable. Notes: JBI questions for cross sectional studies: Q1= Were the criteria for inclusion in the sample clearly defined?; Q2= Were the study subjects and the setting described in detail?; Q3= Was the exposure measured in a valid and reliable way?; Q4= Were objective, standard criteria used for measurement of the condition?; Q5= Were confounding factors identified?; Q6= Were strategies to deal with confounding factors stated?; Q7= Were the outcomes measured in a valid and reliable way?; Q8= Was appropriate statistical analysis used?. JBI questions for quasi-experimental studies: Q1= Is it clear in the study what is the "cause" and what is the "effect" (i.e. there is no confusion about which variable comes first)?; Q2= Was there a control group?; Q3= Were participants included in any comparisons similar?; Q4= Were the participants included in any comparisons receiving similar treatment/care, other than the exposure or intervention of interest?; Q5= Were there multiple measurements of the outcome, both pre and post the intervention/exposure?; Q6= Were the outcomes of participants included in any comparisons measured in the same way?; Q7= Were outcomes measured in a reliable way?; Q8= Was follow-up complete and if not, were differences between groups in terms of their follow-up adequately described and analyzed?; Q9= Was appropriate statistical analysis used?. JBI questions for RCT studies: Q1= Was true randomization used for assignment of participants to treatment groups?; Q2= Was allocation to treatment groups concealed?; Q3= Were treatment groups similar at the baseline?; Q4= Were participants blind to treatment assignment?; Q5= Were those delivering the treatment blind to treatment assignment?; Q6= Were treatment groups treated identically other than the intervention of interest?; Q7= Were outcome assessors blind to treatment assignment?; Q8= Were outcomes measured in the same way for treatment groups?; Q9= Were outcomes measured in a reliable way?; Q10= Was follow up complete and if not, were differences between groups in terms of their follow up adequately described and analysed?; Q11= Were participants analysed in the groups to which they were randomized?; Q12= Was appropriate statistical analysis used?; Q13= Was the trial design appropriate and any deviations from the standard RCT design (individual randomization, parallel groups) accounted for in the conduct and analysis of the trial?. JBI questions for diagnostic test acuracy studies: Q1= Was a consecutive or random sample of patients enrolled?; Q2= Was a case control design avoided?; Q3= Did the study avoid inappropriate exclusions?; Q4= Were the index test results interpreted without knowledge of the results of the reference standard?; Q5= If a threshold was used, was it pre-specified?; Q6= Is the reference standard likely to correctly classify the target condition?; Q7= Were the reference standard results interpreted without knowledge of the results of the index test?; Q8= Was there an appropriate interval between index test and reference standard?; Q9= Did all patients receive the same reference standard?; Q10= Were all patients included in the analysis?. JBI questions for cohort study: Q1= Were the two groups similar and recruited from the same population?; Q2= Were the exposures measured similarly to assign people to both exposed and unexposed groups?; Q3= Was the exposure measured in a valid and reliable way?; Q4= Were confounding factors identified?; Q5= Were strategies to deal with confounding factors stated?; Q6= Were the groups/participants free of the outcome at the start of the study (or at the moment of exposure)?; Q7= Were the outcomes measured in a valid and reliable way?; Q8= Was the follow up time reported and sufficient to be long enough for outcomes to occur?; Q9= Was follow up complete, and if not, were the reasons to loss to follow up described and explored?; Q10= Were strategies to address incomplete follow up utilized?; Q11= Was appropriate statistical analysis used?.

References

28. Moola, S.; Munn, Z.; Tufanaru, C.; Aromataris, E.; Sears, K.; Sfetcu, R.; Currie, M.; Lisy, K.; Qureshi, R.; Mattis, P.; et al. Chapter 7: Systematic Reviews of Etiology and Risk. In *JBI Manual for Evidence Synthesis*; Aromataris, E., Munn, Z., Eds.; JBI: Adelaide, Australia, 2020; pp. 219–272. https://doi.org/10.46658/JBIMES-20-08.
29. Amaefule, C.O.; Lüdtke, S.; Klostermann, A.; Hinz, C.A.; Kampa, I.; Kirste, T.; Teipel, S. At crossroads in a virtual city: Effect of spatial disorientation on gait variability and psychophysiological response among healthy older adults. *Gerontology* **2023**, *69*, 450–463. https://doi.org/10.1159/000527503.
30. Andac, S.; Stolle, F.H.; Bernard, M.; Al-Nosairy, K.O.; Wolbers, T.; Hoffmann, M.B. Navigation performance in glaucoma: Virtual-reality-based assessment of path integration. *Sci. Rep.* **2024**, *14*, 21320. https://doi.org/10.1038/s41598-024-72040-8.
31. Bayahya, A.Y.; Alhalabi, W.; AlAmri, S.H. Smart health system to detect dementia disorders using virtual reality. *Healthcare* **2021**, *9*, 810. https://doi.org/10.3390/healthcare9070810.
32. Cammisuli, D.M.; Isella, V.; Verde, F.; Silani, V.; Ticozzi, N.; Pomati, S.; Bellocchio, V.; Granese, V.; Vignati, B.; Marchesi, G.; et al. Behavioral disorders of spatial cognition in patients with mild cognitive impairment due to Alzheimer's disease: Preliminary findings from the BDSC-MCI project. *J. Clin. Med*. **2024**, *13*, 1178. https://doi.org/10.3390/jcm13041178.
33. Castegnaro, A.; Howett, D.; Li, A.; Harding, E.; Chan, D.; Burgess, N.; King, J. Assessing mild cognitive impairment using object-location memory in immersive virtual environments. *Hippocampus* **2022**, *32*, 660–678. https://doi.org/10.1002/hipo.23458.
34. Castegnaro, A.; Ji, Z.; Rudzka, K.; Chan, D.; Burgess, N. Overestimation in angular path integration precedes Alzheimer's dementia. *Curr. Biol.* **2023**, *33*, 4650–4661.e7. https://doi.org/10.1016/j.cub.2023.09.047.
35. Castillo Escamilla, J.; León Estrada, I.; Alcaraz-Iborra, M.; Cimadevilla Redondo, J.M. Aging: Working memory capacity and spatial strategies in a virtual orientation task. *GeroScience* **2023**, *45*, 159–175. https://doi.org/10.1007/s11357-022-00599-z.
36. Chatterjee, R.; Moussavi, Z. Evaluation of a cognition-sensitive spatial virtual reality game for Alzheimer's disease. *Med. Biol. Eng. Comput.* **2024**, *63*, 1355–1365. https://doi.org/10.1007/s11517-024-03270-1.
37. Chen, Q.; Wu, Z.; Liu, Y.; Han, L.; Li, Z.; Kan, L.G.; Fan, M. SilverCycling: Exploring the impact of bike-based locomotion on spatial orientation for older adults in VR. *Proc. ACM Interact. Mob. Wearable Ubiquitous Technol.* **2024**, *8*, 1–24. https://doi.org/10.1145/3678522.
38. Da Costa, R.Q.M.; Pompeu, J.E.; Moretto, E.; Silva, J.M.; Dos Santos, M.D.; Nitrini, R.; Brucki, S.M.D. Two immersive virtual reality tasks for the assessment of spatial orientation in older adults with and without cognitive impairment: Concurrent validity, group comparison, and accuracy results. *J. Int. Neuropsychol. Soc.* **2021**, *28*, 460–472. https://doi.org/10.1017/s1355617721000655.
39. Silva, J.M.D.; Santos, M.D.D.; Costa, R.Q.M.D.; Moretto, E.G.; Viveiro, L.A.P.; Lopes, R.D.; Brucki, S.M.D.; Pompeu, J.E. Applicability of an immersive virtual reality system to assess egocentric orientation of older adults. *Arq. Neuropsiquiatr.* **2023**, *81*, 19–26. https://doi.org/10.1055/s-0042-1759762.
40. Diersch, N.; Valdes-Herrera, J.P.; Tempelmann, C.; Wolbers, T. Increased hippocampal excitability and altered learning dynamics mediate cognitive mapping deficits in human aging. *J. Neurosci.* **2021**, *41*, 3204–3221. https://doi.org/10.1523/jneurosci.0528-20.2021.
41. Fu, X.; Zhang, Z.; Zhou, Y.; Chen, Q.; Yang, L.-Z.; Li, H. The split-half reliability and construct validity of the virtual reality-based path integration task in the healthy population. *Brain Sci.* **2022**, *12*, 1635. https://doi.org/10.3390/brainsci12121635.

42. Goodroe, S.; Fernandez Velasco, P.; Gahnstrom, C.J.; Wiener, J.; Coutrot, A.; Hornberger, M.; Spiers, H.J. Predicting real-world navigation performance from a virtual navigation task in older adults. *PLoS ONE* **2025**, *20*, e0317026. https://doi.org/10.1371/journal.pone.0317026.
43. Hanert, A.; Schönfeld, R.; Weber, F.D.; Nowak, A.; Döhring, J.; Philippen, S.; Granert, O.; Burgalossi, A.; Born, J.; Berg, D.; et al. Reduced overnight memory consolidation and associated alterations in sleep spindles and slow oscillations in early Alzheimer’s disease. *Neurobiol. Dis.* **2023**, *190*, 106378. https://doi.org/10.1016/j.nbd.2023.106378.
44. Hanyu, H.; Koyama, Y.; Umekida, K.; Watanabe, S.; Matsuda, H.; Koike, R.; Takashima, A. Path integration detects prodromal Alzheimer’s disease and predicts cognitive decline. *J. Alzheimers Dis.* **2024**, *101*, 651–660. https://doi.org/10.3233/jad-240347.
45. Hilton, C.; Miellet, S.; Slattery, T.J.; Wiener, J. Are age-related deficits in route learning related to control of visual attention? *Psychol. Res.* **2019**, *84*, 1473–1484. https://doi.org/10.1007/s00426-019-01159-5.
46. Kalantari, S.; Mostafavi, A.; Xu, B.T.; Lee, A.S.; Yang, Q. Comparing spatial navigation in a virtual environment vs. an identical real environment across the adult lifespan. *Comput. Hum. Behav.* **2024**, *157*, 108110. https://doi.org/10.1016/j.chb.2024.108210.
47. Kim, K.W.; Choi, J.D.; Chin, J.; Lee, B.H.; Choi, J.H.; Na, D.L. Development and preliminary validation of a virtual reality memory test for assessing visuospatial memory. *Front. Aging Neurosci.* **2023**, *15*, 1236084. https://doi.org/10.3389/fnagi.2023.1236084.
48. Koike, R.; Soeda, Y.; Kasai, A.; Fujioka, Y.; Ishigaki, S.; Yamanaka, A.; Takaichi, Y.; Chambers, J.K.; Uchida, K.; Watanabe, H.; et al. Path integration deficits are associated with phosphorylated tau accumulation in the entorhinal cortex. *Brain Commun.* **2023**, *6*, fcad359. https://doi.org/10.1093/braincomms/fcad359.
49. Ladyka-Wojcik, N.; Olsen, R.K.; Ryan, J.D.; Barense, M.D. Flexible use of spatial frames of reference for object–location memory in older adults. *Brain Sci.* **2021**, *11*, 1542. https://doi.org/10.3390/brainsci11111542.
50. Lokka, I.E.; Çöltekin, A. Perspective switch and spatial knowledge acquisition: Effects of age, mental rotation ability and visuospatial memory capacity on route learning in virtual environments with different levels of realism. *Cartogr. Geogr. Inf. Sci.* **2019**, *47*, 14–27. https://doi.org/10.1080/15230406.2019.1595151.
51. Lowry, E.; Puthusseryppady, V.; Coughlan, G.; Hornberger, M. Path integration changes as a cognitive marker for vascular cognitive impairment?—A pilot study. *Front. Hum. Neurosci.* **2020**, *14*, 131. https://doi.org/10.3389/fnhum.2020.00131.
52. McAvan, A.S.; Du, Y.K.; Oyao, A.; Doner, S.; Grilli, M.D.; Ekstrom, A. Older adults show reduced spatial precision but preserved strategy-use during spatial navigation involving body-based cues. *Front. Aging Neurosci.* **2021**, *13*, 640188. https://doi.org/10.3389/fnagi.2021.640188.
53. McCracken, M.K.; Shayman, C.S.; Fino, P.C.; Stefanucci, J.K.; Creem-Regehr, S.H. A Comparison of the effects of older age on homing performance in real and virtual environments. *IEEE Trans. Vis. Comput. Graph.* **2025**, *31*, 3213–3222. https://doi.org/10.1109/tvcg.2025.3549901.
54. Newton, C.; Pope, M.; Rua, C.; Henson, R.; Ji, Z.; Burgess, N.; Rodgers, C.T.; Stangl, M.; Dounavi, M.E.; Castegnaro, A.; et al. Entorhinal-based path integration selectively predicts midlife risk of Alzheimer’s disease. *Alzheimers Dement.* **2024**, *20*, 2779–2793. https://doi.org/10.1002/alz.13733.
55. Noguera, C.; Carmona, D.; Rueda, A.; Fernández, R.; Cimadevilla, J.M. Shall we dance? Dancing modulates executive functions and spatial memory. *Int. J. Environ. Res. Public Health* **2020**, *17*, 1960. https://doi.org/10.3390/ijerph17061960.
56. Oliver, A.; Wildschut, T.; Redhead, E.S.; Parker, M.O.; Sharif, S.; Wood, A.P.; Sedikides, C.; Cheston, R. Benefits of nostalgic landmarks for people living with Alzheimer’s disease. *J. Alzheimers Dis.* **2024**, *102*, 683–702. https://doi.org/10.1177/13872877241291908.

57. Park, J.H. Can the virtual reality-based spatial memory test better discriminate mild cognitive impairment than neuropsychological assessment? *Int. J. Environ. Res. Public Health* **2022**, *19*, 9950. https://doi.org/10.3390/ijerph19169950.
58. Puthus-seryppady, V.; Morrissey, S.; Spiers, H.; Patel, M.; Hornberger, M. Predicting real world spatial disorientation in Alzheimer's disease patients using virtual reality navigation tests. *Sci. Rep.* **2022**, *12*, 13397. https://doi.org/10.1038/s41598-022-17634-w.
59. Qiu, Z.; Ashour, M.; Zhou, X.; Kalantari, S. NavMarkAR: A landmark-based augmented reality (AR) wayfinding system for enhancing older Adults' spatial learning. *Adv. Eng. Inform.* **2024**, *62*, 102635. https://doi.org/10.1016/j.aei.2024.102635.
60. Rinne, K.; Memmert, D.; Bock, O. Proficiency of allocentric and egocentric wayfinding: A comparison of schoolchildren with young adults and older adults. *J. Nav.* **2022**, *75*, 528–539. https://doi.org/10.1017/s0373463321000965.
61. Shayman, C.S.; McCracken, M.K.; Finney, H.C.; Katsanevas, A.M.; Fino, P.C.; Stefanucci, J.K.; Creem-Regehr, S.H. Effects of older age on visual and self-motion sensory cue integration in navigation. *Exp. Brain Res.* **2024**, *242*, 1277–1289. https://doi.org/10.1007/s00221-024-06818-7.
62. Stramba-Badiale, C.; Tuena, C.; Goulene, K.M.; Cipresso, P.; Morelli, S.; Rossi, M.; D'Avenio, G.; Stramba-Badiale, M.; Riva, G. Enhancing spatial navigation skills in mild cognitive impairment patients: A usability study of a new version of ANTaging software. *Front. Hum. Neurosci.* **2024**, *17*, 1310375. https://doi.org/10.3389/fnhum.2023.1310375.
63. Sunami, R.; Nakamoto, T.; Cohen, N.; Kobayashi, T.; Yamamoto, K. Exploring the effects of olfactory VR on visuospatial memory and cognitive processing in older adults. *Sci. Rep.* **2025**, *15*, 10805. https://doi.org/10.1038/s41598-025-94693-9.
64. Tuena, C.; Serino, S.; Pedroli, E.; Stramba-Badiale, C.; Goulene, K.M.; Stramba-Badiale, M.; Riva, G. Embodied spatial navigation training in mild cognitive impairment: A proof-of-concept trial. *J. Alzheimers Dis.* **2024**, *100*, 923–934. https://doi.org/10.3233/jad-240200.
65. Tuena, C.; Serino, S.; Goulene, K.M.; Pedroli, E.; Stramba-Badiale, M.; Riva, G. Bodily and visual-cognitive navigation aids to enhance spatial memory recall in mild cognitive impairment. *J. Alzheimers Dis.* **2024**, *99*, 899–910. https://doi.org/10.3233/jad-240122.
66. Wang, E.H.J.; Lai, F.H.Y.; Leung, W.M.; Shiu, T.Y.; Wong, H.; Tao, Y.; Zhao, X.; Zhang, T.Y.T.; Yee, B.K. Assessing rapid spatial working memory in community-living older adults in a virtual adaptation of the rodent water maze paradigm. *Behav. Brain Res.* **2024**, *476*, 115266. https://doi.org/10.1016/j.bbr.2024.115266.
67. Wen, D.; Liang, B.; Li, J.; Wu, L.; Wan, X.; Dong, X.; Lan, X.; Song, H.; Zhou, Y. Feature Extraction method of EEG signals evaluating spatial cognition of community elderly with permutation conditional mutual information common space model. *IEEE Trans. Neural Syst. Rehabil. Eng.* **2023**, *31*, 2370–2380. https://doi.org/10.1109/tnsre.2023.3273119.
68. Wiener, J.M.; Carroll, D.; Moeller, S.; Bibi, I.; Ivanova, D.; Allen, P.; Wolbers, T. A novel virtual-reality-based route-learning test suite: Assessing the effects of cognitive aging on navigation. *Behav. Res. Methods* **2019**, *52*, 630–640. https://doi.org/10.3758/s13428-019-01264-8.
69. Xu, T.B.; Mostafavi, A.; Boot, W.R.; Czaja, S.; Kalantari, S. Assessing the feasibility, and efficacy of virtual reality navigational training for older adults. *Innov. Aging* **2024**, *9*, igae099. https://doi.org/10.1093/geroni/igae099.
70. Zuo, Y.; Zhou, J. How to help older adults navigate through VR? Effects in outdoor–indoor transition environments. *Int. J. Hum. Comput. Interact.* **2024**, *41*, 8104–8121. https://doi.org/10.1080/10447318.2024.2405276.
